# Cooperative Solvent‐Thermal Interactions Enable Multi‐Material Nanotransfer Printing for Multi‐Dimensional Molecular Profiling

**DOI:** 10.1002/smll.74945

**Published:** 2026-08-08

**Authors:** Tae Wan Park, Seunghee H. Cho, Tae Yeon Kim, Minjoon Kim, Woo‐Youl Maeng, Eun Bin Kang, Jeehyun Hong, Kang‐Jun Baeg, Seung Sae Hong, Sahn Nahm, Jong‐Heun Lee, Yeon Sik Jung, Woon Ik Park

**Affiliations:** ^1^ Department of Materials Science and Engineering Korea University Seoul Republic of Korea; ^2^ Department of Materials Science and Engineering Pukyong National University Busan Republic of Korea; ^3^ Querrey Simpson Institute for Bioelectronics Northwestern University Evanston Illinois USA; ^4^ Department of Materials Science and Engineering Korea Advanced Institute of Science and Technology (KAIST) Daejeon Republic of Korea; ^5^ Graduate School of Semiconductor Materials and Devices Engineering Ulsan National Institute of Science and Technology Ulsan Republic of Korea; ^6^ Department of Semiconductor Engineering Pukyong National University Busan Republic of Korea; ^7^ Department of Materials Science and Engineering University of California Davis California USA; ^8^ RanoM Inc. Research and Development Division Busan Republic of Korea

**Keywords:** crossbar, multi‐material, nanostructures, nanotransfer printing, SERS

## Abstract

Scalable fabrication of sub‐20 nm multi‐material nanoarchitectures remains a key challenge due to material incompatibilities and integration complexity. Here, we introduce a cooperative solvent–thermal nanotransfer printing (ST‐nTP) technique that enables single‐step, high‐resolution patterning of compositionally heterogeneous materials. The process exploits a synergistic interplay between solvent‐induced polymer swelling and thermal‐pressure‐driven densification to achieve selective material transfer through partially open shadow masks. When combined with the directed self‐assembly of block copolymers, ST‐nTP produces hierarchical 3D multi‐metal nanoarchitectures with nanoscale precision and spatial material selectivity. As a functional demonstration, these structures are deployed as tunable surface‐enhanced Raman scattering (SERS) platforms. Distinct plasmonic responses across metal–wavelength combinations facilitate multi‐dimensional molecular profiling, enabling the extraction of complementary vibrational signatures from complex biological analytes such as *E. coli*. This cooperative solvent–thermal nanofabrication strategy expands the design space for integrated nanostructures and offers a versatile route for advanced sensing, diagnostics, and optoplasmonic applications.

## Graphical abstract

Cooperative solvent‐thermal nanotransfer printing (ST‐nTP) exploits the synergistic interplay between solvent‐induced polymer swelling and thermal‐pressure‐driven densification to achieve selective material transfer, enabling scalable single‐step fabrication of 3D hierarchical multi‐material nanoarchitectures with sub‐20 nm resolution for tunable surface‐enhanced Raman scattering (SERS) platforms.

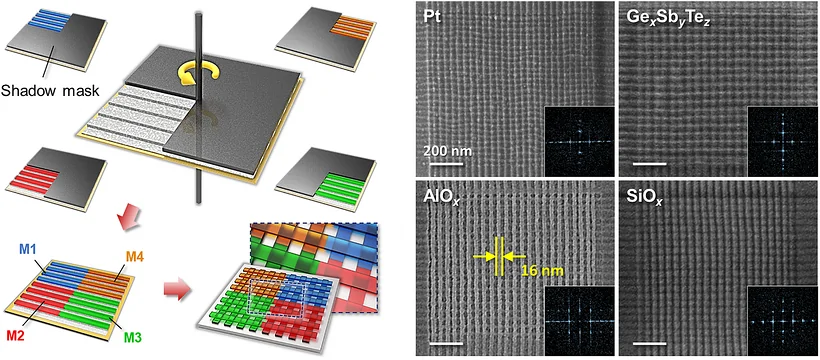

## 1 Introduction

High‐resolution micro‐ and nanoarchitecturing provides a powerful route for tailoring the physical, chemical, and optical properties of materials, enabling a wide range of applications in electronics, photonics, energy devices, and biosensing systems [1, 2, 3, 4, 5, 6, 7, 8, 9]. Micro‐/nano‐structured materials exhibit distinct properties from their bulk counterparts owing to enhanced surface energy and size‐dependent physical phenomena, making them attractive for catalytic, sensing, and optoelectronic applications [10, 11, 12, 13]. For these reasons, various advanced nanofabrication processes, including synthesis and patterning, have been continuously researched and developed [14].

Several advanced nanopatterning technologies based on lithographic processes, such as photolithography [15, 16], extreme ultraviolet lithography (EUV) [17, 18], nanoimprint lithography (NIL) [19, 20, 21], laser lithography [22, 23, 24], and directed self‐assembly (DSA) of block copolymers (BCPs) [25, 26, 27, 28], have been proposed as core technologies for micro‐ to nanoscale fabrication, enabling both device manufacturing and commercial production [29, 30]. These methods can significantly enhance the design and performance of highly integrated circuits and various next‐generation nanodevices utilizing high‐density nanoarrays [31, 32]. Recently, hybrid fabrication strategies that combine multiple patterning mechanisms have been explored to overcome the limitations of individual processes. Representative examples include self‐expansion‐based multipatterning integrated with bottom‐up growth for 2D nanoscale patterning of silicon transistor channels [33] and the combination of two‐photon lithography with femtosecond laser ablation for the fabrication of complex 3D magnetic nanowires [34].

Despite these advances, the fabrication of multi‐material micro‐/nanostructures remains a significant challenge. Most emerging nanofabrication approaches are optimized for individual materials, requiring material‐tailored process conditions and limiting the ability to integrate multiple functional materials within a single architecture. Simultaneous patterning of heterogeneous materials on a 2D surface remains difficult, particularly when high resolution, alignment accuracy, and process compatibility should be maintained. Although new functional materials have significantly enhanced the performance of emerging nanofabrication techniques, as exemplified by highly sensitive ZrO_2_‐BMTST photoresists enabling ultrafast two‐photon lithography (TPL) [35], the development of fabrication strategies capable of accommodating diverse materials remains largely underexplored. Therefore, new nanoarchitecturing methodologies that simultaneously expand both material compatibility and process versatility are required.

Here, we propose a rational nanopatterning strategy to achieve multi‐material patterns in a one‐step process. The solvent‐thermal nanotransfer printing (ST‐nTP) technique, which leverages polymer swelling and densification, enables reliable transfer of 2D and 3D nanoarchitectures. The swollen polymer replica layer, impregnated with solvent vapor, becomes sufficiently soft for transferring functional materials. In addition, the immediate application of uniform pressure and controlled heat to this swollen layer enhances the patternability of functional nanostructures over large areas. The multi‐material pattern on a single replica, achieved through selective deposition using a partially opened shadow mask, can be directly transferred onto arbitrary substrates via the ST‐nTP process, which is an integrated patterning method capable of accommodating multiple materials with varying transfer properties in one operation. Furthermore, a combined patterning strategy that integrates ST‐nTP with the DSA of BCPs enables the generation of 3D hierarchical nanoarchitectures with sub‐20 nm resolution and scalability across different types of materials, including oxides, metals, and semiconductors. We also demonstrate the effectiveness of multi‐metallic nanostructures (Ag, Au, Pt, and Cu) as a surface‐enhanced Raman scattering (SERS) platform for qualitative analysis of both reporter molecules (Rhodamine 6G) and microbiome (*E.coli*) samples. This demonstration highlights significant improvements in the practicality of 3D SERS architectures, paving the way for advanced multi‐material nanoarchitectures with broad potential applications.

## 2 Results and Discussion

### 2.1 Multi‐Material Pattern Formation via ST‐nTP

To fabricate 3D multi‐material patterned structures, we employed a novel solvent‐thermal‐based ST‐nTP process. The key concept of multi‐material pattern formation is the one‐step transfer printing of partially deposited materials onto a single replica pattern. The procedure for the formation of 3D multi‐material crossbar architectures is schematically depicted in Figure 1a. First, a topographically designed replica pattern is created by spin‐coating a polymeric thin film onto a master mold, followed by peeling off the film layer using adhesive polyimide (PI) tape. Prior to the replication step, the master mold is modified to a hydrophobic surface with low surface energy (Figure S1). A reliable replication process requires a well‐defined master mold, which can be fabricated through high‐resolution patterning processes, such as conventional photolithography, NIL, and DSA of BCP [36, 37]. In this study, we utilized a combined lithographic method of photolithography and BCP self‐assembly. This hybrid lithographic strategy offers great potential for achieving high‐resolution and high‐density pattern structures compared to single‐process methods. Additionally, the master mold can be reused semi‐permanently without surface deterioration, as the replication process does not cause any adverse effects, such as heat injection, electrical excitation, or mechanical impact. The master mold maintains its structural integrity without any deformation after multiple uses (Figure S2). Next, four different materials are sequentially deposited onto the replica pattern through multiple physical vapor deposition (PVD) processes using a partially opened steel shadow mask. These functional materials are then transferred from the replica to an arbitrary substrate via the ST‐nTP process. Finally, the remaining polymer replica layer is removed by solvent rinsing with a good solvent that has a similar solubility parameter to the replica material. This approach enables the successful formation of multi‐material pattern architectures on the target substrate, and iterative implementation of this patterning technique allows for the fabrication of 3D crossbar structures.

**FIGURE 1:**
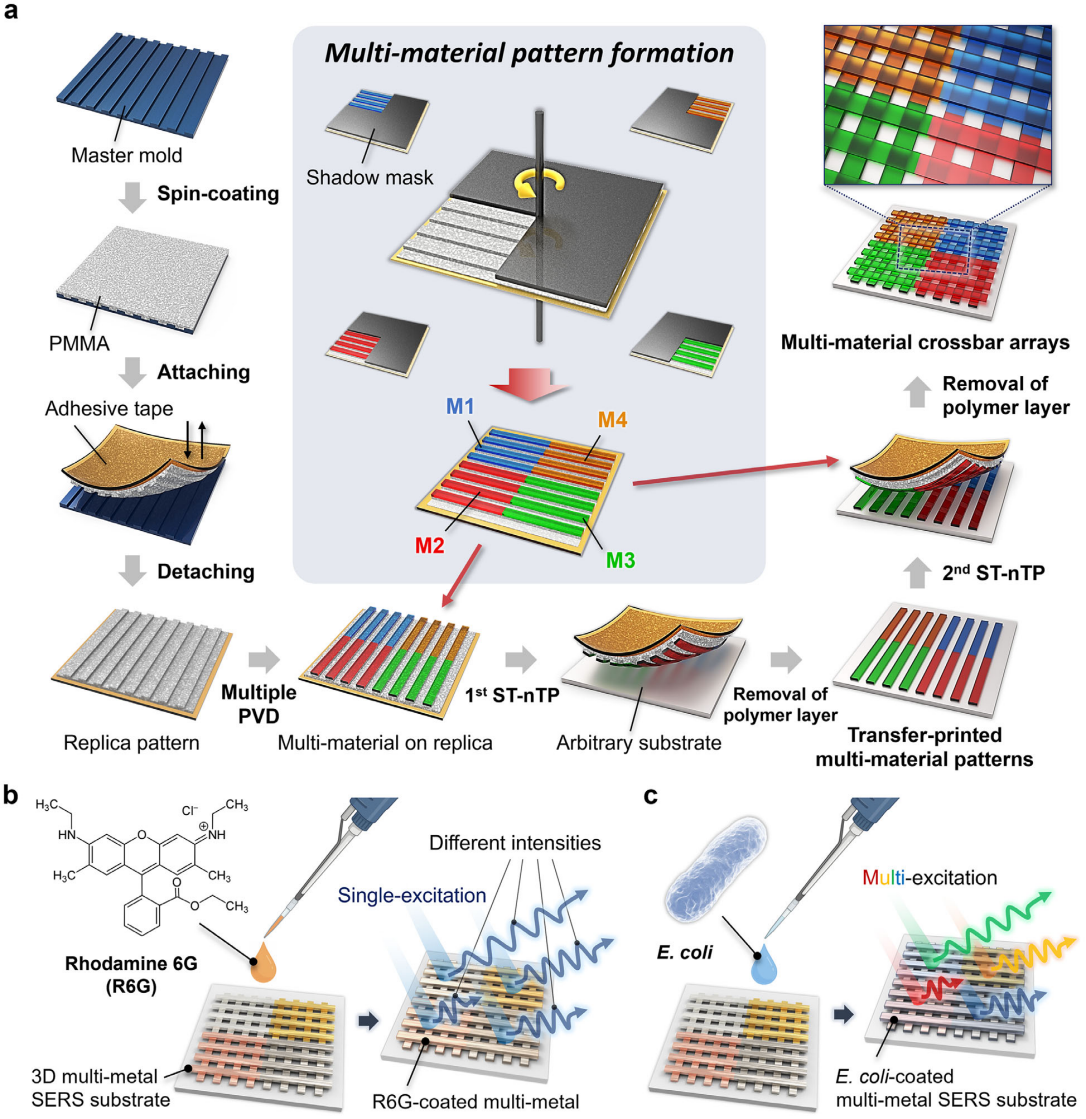
Pattern formation of multi‐material crossbar array for SERS application. (a) Process sequence for fabricating multi‐material pattern structures. The multi‐material nanoarchitectures can be generated through a one‐step transfer printing process that combines area‐selective deposition with the ST‐nTP method. (b) Single‐ and (c) multi‐exitation mode SERS analysis of R6G and *E. coli* utilizing the multi‐material structures. The 3D multi‐metal crossbar nanoarchitecture, fabricated via ST‐nTP, incorporates four distinct metallic materials with unique resonance properties, enabling the collection of exclusive molecular information through multi‐faceted SERS analysis.

Application of multi‐material pattern structures can significantly benefit SERS analysis, which can be demonstrated by single‐ and multi‐excitation modes. Figure 1b shows the single‐excitation mode where the distinctive intrinsic properties of each material, such as Raman cross‐sections and plasmonic efficiencies, result in consistent spectra with varying intensities [38]. Figure 1c shows the multi‐excitation mode, which utilizes the characteristic absorption properties of each material to assign optimal excitation wavelengths [39] to maximize the charge transfer dynamics between the analyte and plasmonic surface for improved Raman cross‐sections [40]. The combination of tailored materials and SERS analysis strategies allows the collection of unique chemical information from each material and thus facilitates comprehensive and qualitative investigation of analytes.

### 2.2 Principle of ST‐nTP

The principle and mechanism of the ST‐nTP process are illustrated in Figure 2. To effectively transfer functional materials from the polymer replica pattern to the target substrate, we exploited the cooperative swelling and densification behavior of the polymeric layer. Specifically, the conceptual understanding of ST‐nTP, which combines polymer swelling during solvent vapor injection and layer densification during uniform heat and pressure activation, is schematically depicted in Figure 2a.

**FIGURE 2:**
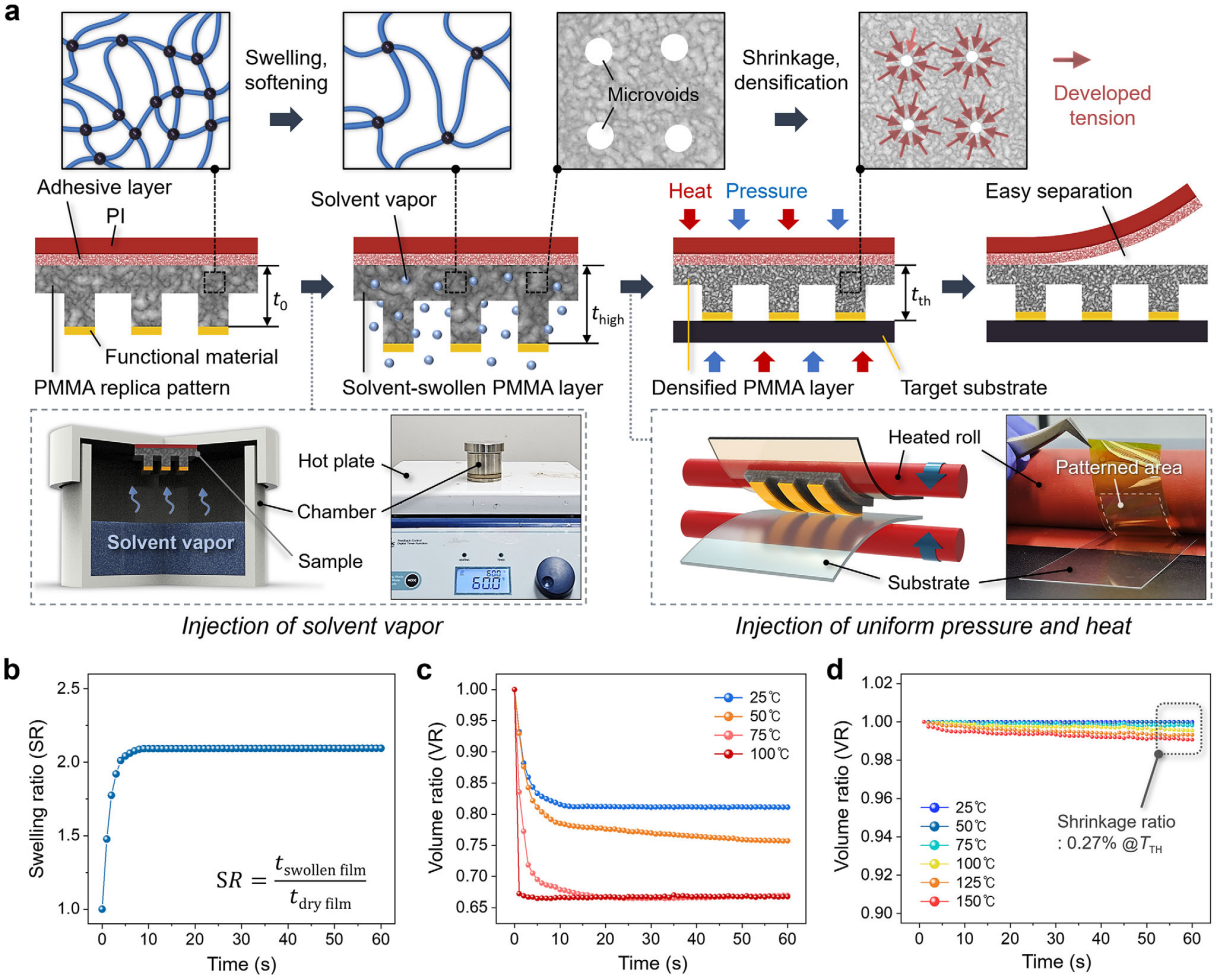
Mechanism of the ST‐nTP process. (a) Schematic illustration of the ST‐nTP mechanism. The functional material is transferred to the target substrate through softening of the PMMA replica layer induced by solvent vapor penetration, followed by densification behavior driven by simultaneous heat‐pressure treatment. (b) Swelling ratio of the PMMA thin film monitored by an in situ reflectometry system. The thickness of the solvent‐saturated PMMA layer increases due to polymer swelling. (c) Volume ratio of the heat‐treated PMMA film at various temperatures, ranging from 25°C to 100°C. (d) Thickness variation of the heat‐injected adhesive layer on PI tape.

As a first step, to initially induce swelling and softening behavior in the polymer film, solvent vapor was infiltrated into the spin‐coated polymer replica layer. In general, the thickness of the polymethyl methacrylate (PMMA) film tends to swell as a result of enhanced chain mobility and enlarged inter‐chain distance upon solvent exposure [41, 42, 43, 44]. Solvent‐induced polymer thin films undergo a reduction in glass transition temperature (*T*_g_), driven by accelerated solvent penetration and plasticization effects, ultimately reducing the entanglement density and softening the film as its mechanical strength diminishes [45, 46]. In addition, solvents with a similar solubility parameter to PMMA are effective for polymer swelling [46, 47, 48]. In this study, solvent vapor generated from a 1:1 mixed solvent of toluene and acetone was injected into the PMMA layer under warm temperature conditions (∼ 60°C), resulting in swelling of the replica film. The sample was placed on top of a steel chamber after sufficient annealing of the solvent (> 3 min) in a heated chamber body using a hot plate. As shown in Figure 2b, the swelling ratio (SR) of the PMMA film, defined as the swollen film thickness divided by the dry film thickness, increased rapidly after solvent injection, reaching 2.09 times the original thickness after 9 s. This result confirms that toluene and acetone molecules from the solvent vapor migrate into the spin‐coated PMMA layer, causing the film to expand (*t*_0_< *t*_high_).

Another advantage of this step in facilitating the transfer process is that the adhesive strength of PI tape is weakened by solvent molecules released from the solvent‐saturated polymer layer. Previous studies have also demonstrated the transferability of solvent‐impregnated soft polymer films [49]. To determine this effect, we compared the adhesion properties of pristine PI tape and solvent‐injected PI tape (see details in the Experimental section). The adhesion strength slightly decreased from 4.38 N to 2.77 N after solvent injection, as shown in Figure S3. Such weakened adhesion, arising from the softening of the solvent‐saturated polymer layer, facilitates the release of replica materials from the adhesive tape to the target substrate. While this soft transfer printing strategy is highly effective for successful pattern transfer, it still requires additional mechanisms to enable reliable large‐area patterning. The conventional nTP method, which relies solely on solvent vapor, is susceptible to process errors caused by excessive solvent exposure and exhibits limited scalability for large‐area patterning, as shown in Figures S4 and S5.

To mitigate these limitations of the solvent‐based approach, we incorporated an additional thermal activation step following the solvent vapor exposure. After bringing the functional material and target substrate into full contact, heat and pressure are simultaneously applied to the swollen PMMA layer by means of a heat‐rolling press system. The thickness of the solvent‐swollen PMMA layer rapidly decreases upon heat treatment (*t*_high_ > *t*_th_). Figure 2c shows the temperature dependency of the volume ratio of heat‐induced PMMA film shrinkage, monitored by an in situ reflectometry system from 25°C (RT) to 100°C. The thickness of the heat‐treated PMMA layer decreased by approximately 19% at RT, 24.3% at 50°C, and 33.6% at 75°C and 100°C after 60 s. This result reflects the recovery behavior of the film when solvent injection is stopped. It should be noted that the process for large‐area patterning was established using an integrated heat activation method compared to the approach relying solely on solvent vapor injection, as shown in Figure S5. The surface of the PMMA thin film heated at various temperatures exhibited microcracks due to the tension developed in the PMMA layer, as shown in Figure S6. These results clearly show that the swollen PMMA layer contracts rapidly through densification during thermal shrinkage. They also imply that a rapid patterning process is essential for producing high‐quality pattern structures via ST‐nTP, as the PMMA layer shrinks quickly when the temperature increases.

Figure S7 shows the pattern transfer yield at various temperatures with a transfer time of three seconds, demonstrating that the threshold temperature (*T*_TH_) for successful patterning using the ST‐nTP approach is approximately 75°C. The mean values and error bands of transfer yields for each sample under various temperatures were quantitatively calculated using ImageJ software. The shrunken and densified PMMA layers induced by heat treatment are shown in Figure S8. The thickness of spin‐coated PMMA decreased by approximately 6.8% when heat was applied at 75°C, regardless of solvent injection, clearly showing the densification of the PMMA layer. This result is attributed to the porous structure developed in the interior of the spin‐coated soft polymer film, which arises from the water content in the coating solution, ambient humidity, and residual solvent retained in the film after the coating process [41, 50]. Figure S9 presents the key parameters determining the *T*_TH_ required for successful pattern transfer. First, the effect of solvent type was evaluated while maintaining a constant PMMA replica thickness and solvent vapor exposure time (36 s). As shown in Figure S9a, toluene and acetone vapors resulted in lower *T*_TH_ values than EtOH and DI water, indicating more effective penetration and softening of the PMMA replica layer due to their similar solubility parameter. Second, the effect of polymer thickness during thermal activation was investigated. A *T*_TH_ of 75.5°C was achieved for a 220 nm thick film, whereas thicker films required higher *T*_TH_ values for successful pattern transfer (Figure S9b). These findings indicate that both solvent selection and replica thickness are critical parameters in governing the *T*_TH_ in the ST‐nTP process.

To further investigate factors affecting thickness variations, we measured the thickness of the adhesive layer on PI tape at various temperatures from 25°C to 150°C (Figure 2d). The maximum shrinkage ratio was nearly 0.93% at 150°C. At the *T*_TH_ of 75°C for successful patterning, the shrinkage ratio was only 0.27%, indicating that the thickness of the adhesive layer does not significantly affect the patternability of the replica pattern.

Additionally, adhesion strength decreases slightly not only due to solvent injection, as mentioned above, but also when heat is applied to adhesive tape. The peel strength of PI tape decreased from 4.37 to 3.33 N when heat was applied between the PI layer and the SiO_2_/Si substrate, as shown in Figure S10. The rapid densification of polymer chains during heat treatment can also weaken adhesion between the adhesive and PMMA layers. This thermal‐pressure‐based approach builds upon our previously established results [36]. In a single thermally assisted nanotransfer printing (T‐nTP) process, a heat injection above 150°C was essential for successful patterning. Under similar conditions, the *T*_TH_ of ST‐nTP is dramatically reduced from 150°C to 75°C, a result that directly stems from the solvent vapor treatment prior to thermal treatment, as shown in Figure S7b. Note S1 and Table S1 detail the respective limitations of previously reported nTP methods and explain how ST‐nTP overcomes them. The ST‐nTP process, driven by a cooperative solvent‐thermal interaction mechanism, enables the polymer replica layer to be easily released from the adhesive tape at warm temperatures (∼75°C), thereby facilitating the transfer of the functional pattern structures onto the surface of the target substrate. This hybrid strategy overcomes the limitations of single solvent‐ and thermal‐based transfer printing methods, resulting in reliable pattern formation over a large area. A new transfer printing process with a *T*_TH_ of 75°C not only can be extensively extended to a wide range of materials, including water‐soluble polymers, biocompatible polymers, and functional luminescent and semiconductor organic materials that are sensitive to high temperatures, but also opens a promising pathway for reliable applications in flexible electronics, bioprinting, and display technologies.

### 2.3 3D Nanoarchitectures With Sub‐20 nm Resolution

Figure 3 shows various nanoarchitectures transfer‐printed by ST‐nTP. To generate sub‐20 nm high‐density nanostructures, we employed a combined mold fabrication strategy using conventional photolithography and DSA of BCPs, as shown in Figure S11. A cylinder‐forming poly(styrene‐*b*‐dimethylsiloxane) (PS‐*b*‐PDMS) BCP with a molecular weight (MW) of 45.5 kg mol^−1^ (*f*_PDMS_ =  33.7%) was used to fabricate ultrafine SiO*_x_* line structures on a Si mold prepared through photolithography process [51, 52]. Figure S12 shows self‐assembled SiO*_x_* line structures, which are regularly defined with widths and spaces of 16 nm within the Si guiding template. The pattern quality of the self‐assembled line structures was analyzed by grazing‐incidence small‐angle X‐ray scattering (GISAXS) measurement, which demonstrated excellent pattern uniformity, as shown in Figure S12c,d.

**FIGURE 3:**
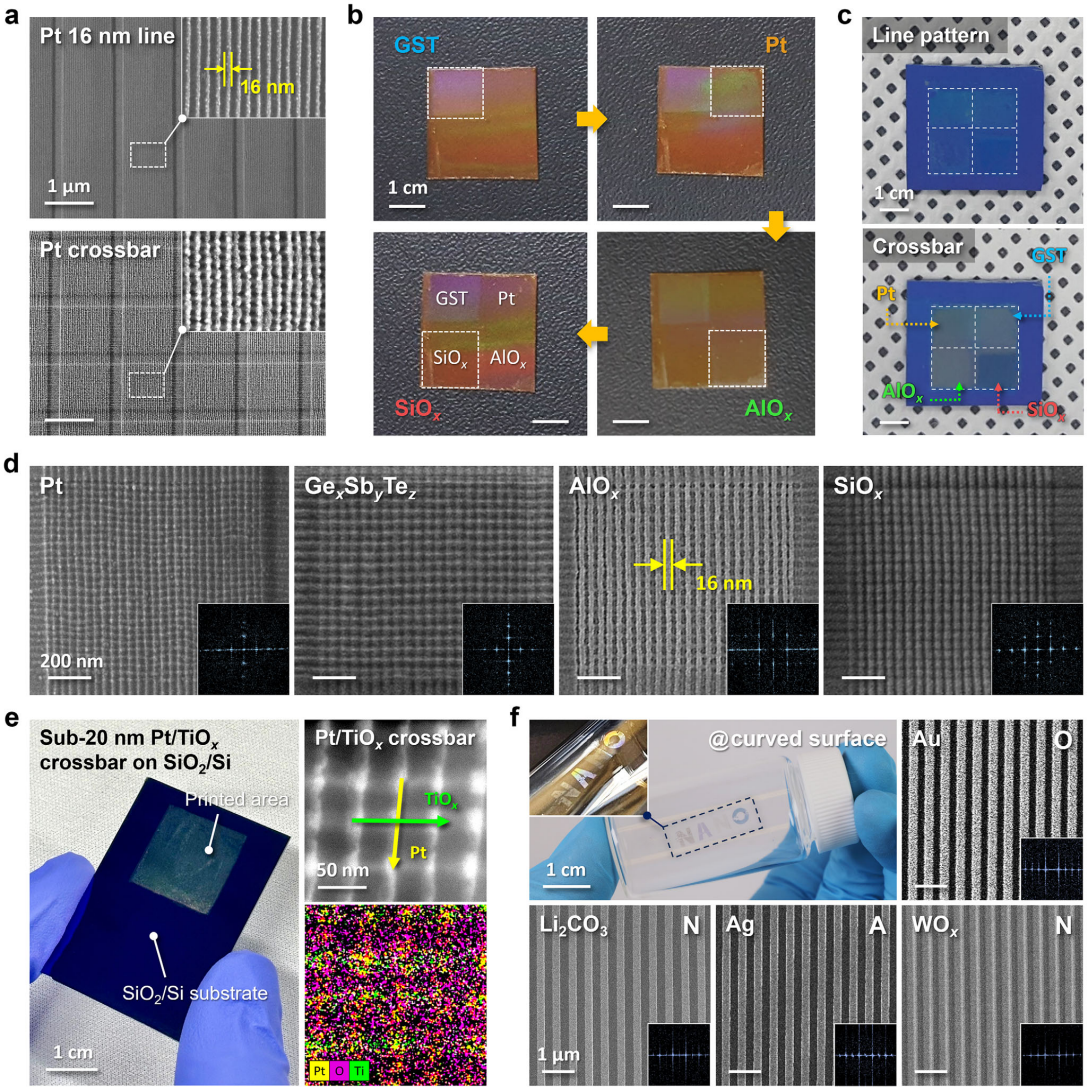
Transfer‐printed various 3D nanoarchitectures via ST‐nTP. (a) Sub‐20 nm Pt line and crossbar patterns fabricated through the ST‐nTP process. (b) Photographic images of replica patterns formed by area‐selective deposition of four materials. (c) Transferred line and crossbar patterns on a SiO_2_/Si substrate. (d) Crossbar patterns with a width of 16 nm, consisting of Pt, Ge*_x_* Sb*_y_* Te*_z_*, AlO*_x_*, and SiO*_x_*, successfully transferred onto a SiO_2_/Si substrate using ST‐nTP. The insets show fast Fourier transform (FFT) patterns obtained from each corresponding SEM image. (e) TiO*_x_*/Pt crossbar nanoarchitecture: (Left) photographic image of the printed patterns, (top right) top‐view bright‐field transmission electron microscopy (TEM) image, and (bottom right) TEM‐EDS elemental mapping image. (f) Transfer‐printed nanopatterns in text shapes (“N,” “A,” “N,” and “O”) composed of various oxide materials (Li_2_CO_3_ and WO*_x_*) and metallic materials (Ag and Au).

High‐resolution Pt nanowires and crossbar arrays with widths of 16 nm were successfully obtained using self‐assembled SiO*_x_* line structures as a master mold, as shown in Figure 3a. Figure 3b displays the replica patterns, where Ge*_x_* Sb*_y_* Te*_z_* (GST), Pt, AlO*_x_*, and SiO*_x_* were sequentially deposited. Using this deposited replica pattern, functional pattern structures were printed onto the SiO_2_/Si substrate. Figure S13 shows the transfer‐printed pattern structures via the cooperative solvent‐thermal transfer printing process. All printed patterns exhibited a reliable line width distribution and a great process window closely resembling that of the master mold, which features sub‐20 nm line widths, as shown in Figure S13a,b. For an additional quantitative validation of the quality of printed patterns, we additionally implemented the power spectral density (PSD) analysis that can show the spatial frequency‐dependent characteristics of line edge roughness (LER) and line width roughness (LWR). Figure S13c,d presents the PSD curves for the LWR and LER of self‐assembled nanomold and transfer‐printed functional nanostructures (Pt, GST, AlO*_x_*, and SiO*_x_*), indicating the similarity of the pattern structures across various spatial frequencies. The geometry and LER of transfer‐printed nanostructures with sub‐20 nm resolution are primarily determined by the fidelity of the original block copolymer template, demonstrating faithful replication of the template‐defined pattern characteristics. Figure S14 illustrates the deposition strategy based on the line directions of two distinct replica patterns. To obtain a crossbar structure, a second replica pattern must be deposited with a 90° rotation relative to the first replica pattern to ensure proper alignment. Figure 3c,d shows hierarchically stacked 3D nanoarchitectures formed by the ST‐nTP process using various materials, including metallic (Pt), insulating (AlO*_x_*), and semiconducting (GST and SiO*_x_*) materials. The inset fast Fourier transform (FFT) patterns from each scanning electron microscope (SEM) image clearly indicate highly ordered crossbar architectures.

Figure 3e and Figure S15 show dark‐field transmission electron microscopy (TEM) and energy‐dispersive spectrometry (EDS) elemental mapping images of a TiO*_x_*/Pt crossbar array with a line width of 16 nm on the SiO_2_/Si substrate. Notably, these results demonstrate that the ST‐nTP method enables reliable pattern formation with multiple materials on target substrates. The 3D hierarchically stacked nanoarchitectures retained their structural stability without any delamination or damage following an ultrasonic delamination test (Figure S16). Figure 3f and Figure S17 highlight the high applicability of the ST‐nTP process for pattern formation with various shapes and materials. Specifically designed shadow masks were used to sequentially deposit various shapes (N:Li_2_CO_3_, A: Ag, N: WO*_x_*, and O: Au) on the replica pattern and transferred onto a curved glass vial substrate. The selectively transfer‐printed alphabetical shapes exhibited slight variations in luminance depending on the refractive index of the materials, despite having the same deposited thickness. These results underscore that the new ST‐nTP process enables reliable pattern transfer with sub‐20 nm resolution and high material scalability. On the basis of these findings, further development of this ST‐nTP strategy toward conformal patterning on curved surfaces could extend its applicability across diverse advanced nanomanufacturing platforms.

### 2.4 Surface‐Enhanced Raman Spectroscopy (SERS)

To assess the performance of the multi‐material 3D nanostructures as SERS‐active platforms, we fabricated crossbar arrays composed of plasmonic metals. Such 3D crossbar arrays can produce strong surface‐enhanced plasmon resonance compared to single‐layer nanostructures or thin films of metal due to the highly dense plasmonic “hot spots” formed between stacked layers (Figure S18). Silver (Ag), gold (Au), platinum (Pt), and copper (Cu) were selected to explore a broad range of plasmonic and chemical behaviors. Specifically, Ag and Au support strong plasmon resonance, while Au is more chemically stable. Pt and Cu can output unique vibrational signals owing to their distinctive absorption properties. The nanoarchitectures were produced using our rotating shadow‐mask lithography process, which enables precise spatial control over nanoscale patterning across multiple materials. This technique ensured uniform structural features across the different regions, as evidenced by the top‐view SEM images in Figure 4a and supplementary data in Figure S19. The morphological features such as line‐edge roughness and breaks in bars each arise from the fabrication method of the master mold and intentional control of deposition rate for in‐plane SERS enhancement, respectively.

**FIGURE 4:**
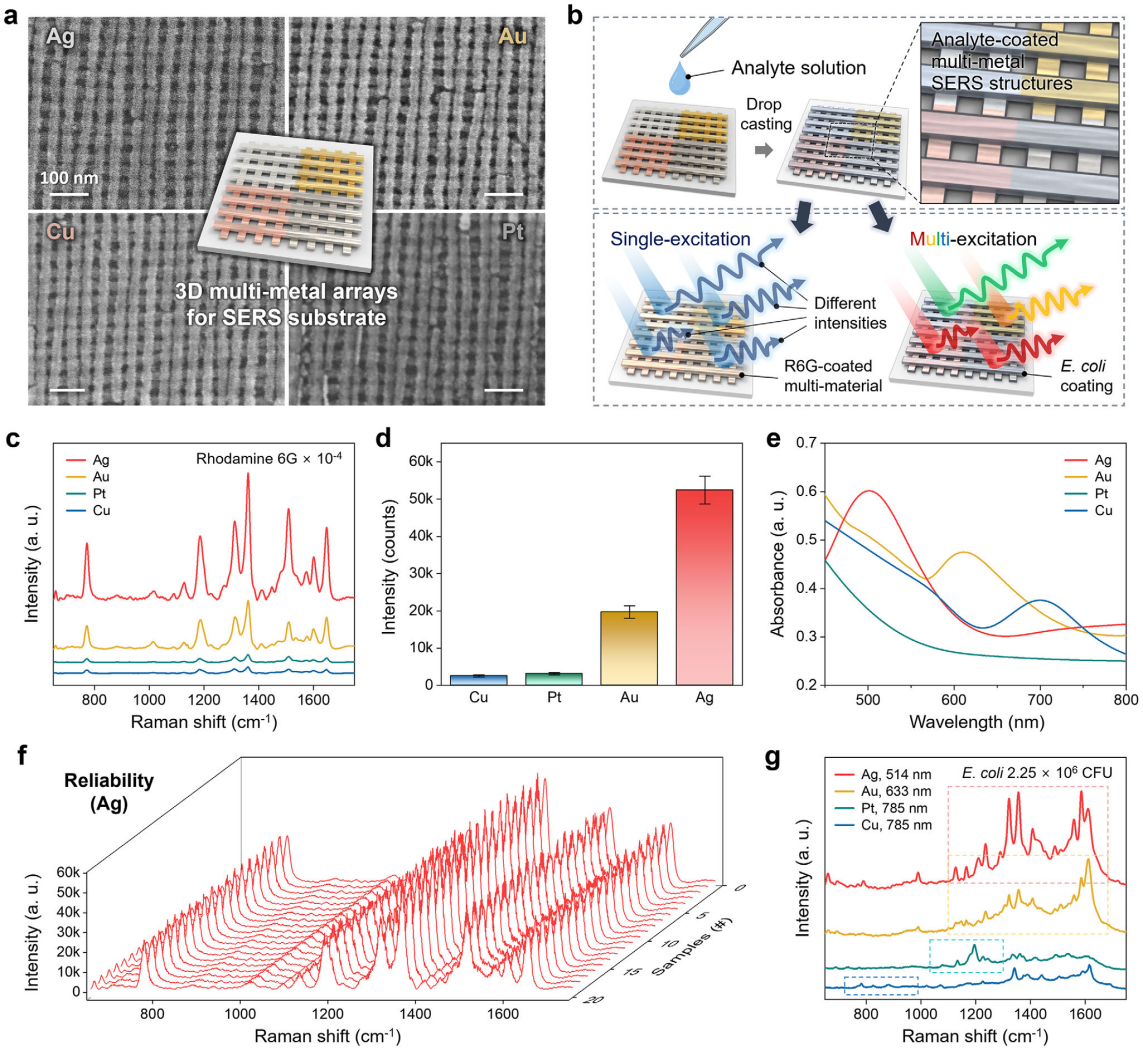
SERS analysis using 3D multi‐metal arrays. (a) Top‐view SEM images of Ag, Au, Pt, and Cu 3D multi‐metal crossbar arrays. Scale bar, 200 nm. (b) Schematic illustration of SERS analysis process flow. Analyte preparation followed by single‐ or multi‐excitation mode SERS analysis. (c) Single‐excitation SERS spectra from 10^−4^ M R6G by material, Ag, Au, Pt, and Cu. (d) Mean SERS intensity at 1361 cm^−1^ obtained from 20 different points. (e) Absorption spectra of 3D multi‐metal array at different material regions. (f) Graph arrays showing SERS spectra from R6G, obtained from 20 points for Ag material. (g) SERS spectra of *E. coli* obtained from different material regions using various excitation wavelengths. Highlighted regions in the graph indicate exclusive information from each spectrum.

The general SERS measurement workflow is depicted schematically in Figure 4b. The analytes used in this study were Rhodamine 6G (R6G), a standard Raman reporter molecule, and *Escherichia coli* (*E. coli*), a representative model organism widely used in microbial sensing studies. Each analyte solution with a known volume was drop‐cast onto the 3D multi‐metal arrays to form a thin film to facilitate strong contact between the analyte and the plasmonic surface and also enable accurate calculation of the enhancement factors. SEM analysis after each step for preparation and analysis shows that the 3D crossbar architectures maintain structural stability (Figure S20). The SERS characterization was then performed using both single‐ and multi‐excitation modes.

In the single‐excitation SERS mode (λ = 514 nm), distinct spectral signatures of R6G were acquired from each of the four material regions. As shown in Figure 4c, all materials produced identifiable Raman peaks, although with varying intensities. The peak at 1361 cm^−^^1^, characteristic of R6G, was used for quantitative comparison across materials. Figure 4d summarizes the mean SERS intensities at this vibrational mode, averaged over 20 randomly selected points per material, revealing Ag and Au as the most efficient SERS substrates under this excitation. Pt and Cu exhibited lower intensities, reflecting their weaker plasmonic resonance or less favorable energy alignment with the excitation wavelength. Enhancement factor values were calculated as 1.24 × 10^7^, 4.71 × 10^6^, 7.59 × 10^5^, and 6.23 × 10^5^, for Ag, Au, Pt, and Cu, respectively, using 10^−9^ M R6G (see Figure S21 and Note S2 for details). Such high SERS enhancement properties are valuable in multiplexing analytes with varying Raman cross‐sections, as demonstrated using TAMRA (5‐Carboxytetramethylrhodamine) and cortisol molecules (Figure S22).

To further understand the observed SERS performance differences, we analyzed the optical absorption characteristics of the nanostructures. Figure 4e presents the absorption spectra for each metal region. Ag, Au, and Cu exhibit distinct plasmonic resonance peaks centered at approximately 502, 611, and 700 nm, respectively. These values align with their expected localized surface plasmon resonance (LSPR) behavior. Unlike Ag, Au, and Cu, Pt exhibits a largely featureless absorption spectrum over the measured wavelength range, indicating negligible plasmonic resonance and making it a suitable reference for evaluating the contribution of electromagnetic enhancement.

Reproducibility is a critical requirement for practical SERS applications. Multiple SERS spectra collected from 20 different points on each material region displayed consistent spectral characteristics and peak patterns (Figure 4f and Figure S23) with relative standard deviations of < 10% (Figure S24), confirming uniform field enhancement over a centimeter‐scale area.

In the multi‐excitation mode SERS experiments, we used three different laser wavelengths—514, 633, and 785 nm—to interrogate the *E. coli* samples across the four metal regions. The resulting spectra, shown in Figure 4g, reveal material‐ and wavelength‐specific variations in spectral features. Ag and Au, using excitation wavelengths of 514 and 633 nm, show the strongest signal and the greatest number of characteristic Raman bands that represent *E. coli* owing to its sharp plasmon resonance. Pt and Cu, using an excitation wavelength of 785 nm, displayed unique vibrational features not found in the spectra of Ag and Au, such as 827, 1195, and 1342 cm^−1^. Spectra with detailed peak labels (Figure S25) and a full list of the peaks are provided in Table S2. The shaded regions in Figure S25 indicate the standard deviation of each spectrum, demonstrating the reproducibility of each *E. coli* spectrum (Figure S26). Such variations can be attributed to the complex and heterogeneous extracellular composition of *E. coli*, which leads to nonuniform areal distribution in molecular components within the small laser spot area.

The emergence of unique Raman peaks under specific plasmonic material–wavelength combinations can be attributed to the interplay of electromagnetic and chemical enhancement mechanisms. When the excitation wavelength closely matches the localized surface plasmon resonance (LSPR) of a given plasmonic material, the resulting near‐field enhancement selectively amplifies vibrational modes that overlap both spatially and spectrally with the local field distribution [53]. In addition, charge‐transfer interactions between the analyte and plasmonic surface can modulate the Raman cross‐section of specific modes, leading to further spectral differentiation across materials [54]. As a result, distinct vibrational features become active only under specific plasmonic material and wavelength conditions, enabling multidimensional molecular profiling.

These findings illustrate the utility of a spatially multiplexed SERS platform composed of diverse plasmonic materials. By leveraging the complementary optical properties of each metal and combining them with multi‐wavelength excitation, our system enhances spectral diversity and information richness. This enables more comprehensive molecular fingerprinting of complex biological analytes, such as bacteria, and supports the development of adaptive molecular libraries for multiplexed sensing applications.

## 3 Conclusion

In summary, we have proposed a simple yet highly effective nanofabrication method that simultaneously generates multi‐material nanoarchitectures through a one‐step process. The ST‐nTP process, which utilizes two distinct polymer behaviors swelling and densification was developed to effectively transfer multi‐material patterns onto arbitrary substrates. The nanoscale pattern structures were easily transferred at low temperatures through the application of both uniform pressure and gentle heat, owing to the solvent‐impregnated soft polymer replica layer. The ST‐nTP process substantially reduced the *T*_TH_ for successful large‐area patterning to half that of previously reported transfer printing methods. High‐resolution 3D nanoarchitectures with sub‐20 nm resolution and multiple materials, such as Pt, GST, AlO*_x_*, and SiO*_x_*, were obtained through a combined fabrication method of ST‐nTP and DSA of BCP, successfully produced on both flat SiO_2_/Si and curved vial substrates. Furthermore, we demonstrated how to enhance the practicality of 3D multi‐metal arrays in SERS by fine‐tuning their plasmonic properties with distinct resonance characteristics in analyte‐coated materials across multiple excitation wavelengths. This capability enables multi‐dimensional molecular profiling, offering access to richer spectral information from complex analytes such as *E. coli*. In particular, utilization of various plasmonic materials may diversify approaches for microbiome analysis. For example, biocompatible metals such as Au may facilitate non‐invasive investigation of extracellular components, and metals with antibacterial properties can induce cell lysis to expose intracellular components for analysis. The findings of this study are expected to provide potential pathways for developing various micro‐ to nanoscale fabrication technologies applicable to state‐of‐the‐art devices in the near future. The demonstrated multi‐material crossbar fabrication strategy also provides a foundation for future development of single‐pattern SERS platforms incorporating multiple metals, enabling spatially multiplexed sensing with distinct plasmonic and chemical enhancement characteristics.

## 4 Experimental Section

### 4.1 Fabrication of a Master Mold with Sub‐20 nm Line Structures

To obtain an ultrafine nanomold, a combined patterning method of photolithography and DSA of BCP was used. First, a Si guiding template was fabricated using conventional KrF photolithography, followed by a reactive ion etching (RIE) process. After spin‐coating the positive photoresist (PR, Dongjin Semichem Co. Ltd.) with a thickness of 400 nm on an eight‐inch Si wafer, the PR film was exposed using a KrF scanner (Nikon, NSR‐S203B) and then developed using a developer solution (tetramethylammonium hydroxide, Dongjin Semichem Co. Ltd.). The remaining PR patterns were used as an etch mask to engrave the pattern structure with CF_4_ plasma using RIE (working pressure: 9 mTorr, plasma power: 180 W). Second, the BCP self‐assembly process was performed on the Si trench fabricated through the photolithography process. The surface of the topographically designed Si pattern was treated with a hydroxy‐terminated polystyrene (PS‐OH) homopolymer with an MW of 38 kg/mol to promote the DSA of BCP. A Si‐containing, cylinder‐forming PS‐*b*‐PDMS BCP (*f*_PDMS_ =  33.7% and MW = 45.5 kg/mol) was used to generate well‐defined sub‐20 nm line structures. This BCP material, dissolved in toluene solvent at a concentration of 1.0 wt%, was spin‐cast onto the surface‐patterned Si guiding template and annealed in a binary solvent mixture of toluene and heptane at 85°C for 60 min. During the self‐assembly of PS‐*b*‐PDMS BCP, PDMS preferentially segregates to the air/polymer interface because of its lower surface energy than that of PS, thereby forming a PDMS‐rich surface layer (top‐PDMS) on the surface of the BCP film [55]. The annealed BCP sample was then etched by CF_4_ plasma (gas flow rate: 30 standard cubic centimeters per minutes (sccm), working pressure: 15 mTorr, plasma power: 60 W, etching time: 15 sec) and O_2_ plasma (gas flow rate: 30 sccm, working pressure: 15 mTorr, plasma power: 60 W, etching time: 36 sec) to remove the top PDMS and the PS matrix, respectively, resulting in self‐assembled SiO*_x_* (oxidized PDMS) line structures with a width and space of 16 nm.

### 4.2 Pattern Replication

A surface‐patterned master mold was modified with a hydroxy‐terminated polydimethylsiloxane (PDMS‐OH, Polymer Source Inc.) brush at 150°C for 1.5 h in a vacuum oven. The brush‐treated Si surface facilitates the easy separation of the polymer replica material from the mold by lowering the surface energy compared to that of the replica material [56, 57]. The polymer replica material, dissolved in a good solvent, was spin‐coated onto the master mold at 5000 rpm for 22 sec, resulting in a soft polymer thin film. This coated layer was then peeled off using adhesive PI tape. A rolling‐press system was used to apply uniform pressure across all surface areas to be replicated during the process of peeling off the replica layer from the master mold.

### 4.3 Measurement of Polymer Thickness

The thicknesses of swollen PMMA, shrunken PMMA, and shrunken adhesive layers were measured using a reflectometer (F20‐UV, Filmetrics Inc.) with a wavelength range of 350 to 1100 nm. All measured variable layers were calculated by dividing the measured thickness by their respective initial thicknesses.

### 4.4 Simultaneous Activation of Uniform Heat and Pressure

A heat‐rolling press system (LAMIART‐470 LSI, GMP Corp.), equipped with four rolls encapsulated by elastic silicone rubber, was used for the uniform activation of heat and pressure. This system provides both uniform heat transfer and pressure treatments by controlling the roll gap, printing temperature, and rotation speed. The rolling speed was set to 770 mm/min to quickly inject both heat and pressure into the patternable area.

### 4.5 Peel Strength Measurement

The peel strength test was conducted using an advanced force gauge (M5‐05, Mark‐10) equipped with a motorized stage (ESM303, Mark‐10). An adhesive PI tape was peeled off from the target substrate with an angle of 90° at a speed of 45 mm/min.

### 4.6 SERS Analysis

Raman scattering signals were collected using a dispersive Raman microscope (ARAMIS, Horiba) system including excitation lasers with wavelengths of λ = 514, 633, and 785 nm. Gratings with 1200 grooves per 1 mm, a 50× microscope, and a beam size of 1 µm in diameter were used. A data acquisition time of 0.5 s was applied for both R6G and *E. coli* measurements. All spectra were processed to remove major fluorescence backgrounds with baseline correction. For preparation of R6G, 20 µL of an ethanol solution with a concentration of 10^−4^ M was drop‐cast onto the substrate. To prepare cell analytes, *Escherichia coli* (*E. coli*, New England Biolabs) was stored in a mixture of glycerol and broth solutions prior to growth. 0.5 mL of the mixture was added to 50 mL of broth for overnight incubation, followed by 1 mL transfer to 50 mL fresh broth for 4 h incubation. All incubations were performed at 37°C. Resulting solutions were centrifuged once, then twice in deionized water to remove both solutions. 5 µL of the final cell solution was drop‐casted onto the substrate. Prior to Raman measurements of *E. coli*, cells were identified under the microscope, and the laser was focused on locations containing bacterial cells to acquire representative SERS spectra.

### 4.7 Characterization

Ultra‐high‐resolution nanoarchitectures were observed using a field emission SEM (TESCAN MIRA3) at an acceleration voltage of 5–10 kV and a working distance of 3–5 mm. The TiO*_x_*/Pt 16 nm crossbar architecture was examined using TEM‐EDS (JEOL JEM‐ARM200F) operated at 200 kV. The Top‐view TEM sample was prepared by mechanical polishing, followed by ion milling with an Ar ion.

## Author Contributions

T.W.P. and W.I.P. conceived the concept of the research. T.W.P. and T.Y.K. investigated the mechanism of the ST‐nTP method. T.W.P., S.H.C., and M.K. participated in the fabrication of samples. T.W.P. and W.‐Y.M. contributed to adhesion strength measurements. T.W.P. and E.B.K. performed large‐area patterning. S.H.C. and M.K. conducted SERS analysis. J.H. performed GISAXS measurements. T.W.P. and S.H.C. analyzed the data. K.‐J.B., S.S.H., S.N., and J.‐H.L. provided useful insights and critical comments on this project. T.W.P., Y.S.J., and W.I.P. supervised the project. T.W.P., S.H.C., Y.S.J., and W.I.P. wrote the manuscript. All authors discussed the results and commented on the manuscript at all stages.

## Conflicts of Interest

The authors declare no conflict of interest.

## Supporting information




**Supporting File**: smll74945‐sup‐0001‐SuppMat.docx.

## Data Availability

The data that supports the findings of this study are available in the supplementary material of this article.
