# Supplementary material for: Cooperative Solvent‐Thermal Interactions Enable Multi‐Material Nanotransfer Printing for Multi‐Dimensional Molecular Profiling
Source: Small. 2026 Aug 8;22(55):e74945. doi: 10.1002/smll.74945 (PMC13630406; doi:10.1002/smll.74945)
Supplement: Supplementary file 1 [file SMLL-22-e74945-s001.docx]

Supporting Information

Cooperative solvent-thermal interactions enable multi-material nanotransfer printing for multi-dimensional molecular profiling

Tae Wan Park^1,2,3,9^, Seunghee H. Cho^3,4,9^, Tae Yeon Kim^3^, Minjoon Kim^4^, Woo-Youl Maeng^3^, Eun Bin Kang^2,5^, Jeehyun Hong^4^, Kang-Jun Baeg^6^, Seung Sae Hong^7^, Sahn Nahm^1^, Jong-Heun Lee^1,10^, Yeon Sik Jung^4^*, and Woon Ik Park^2,7,8^*

^1^Department of Materials Science and Engineering, Korea University, Seoul 02841, Republic of Korea

^2^Department of Materials Science and Engineering, Pukyong National University, Busan 48513, Republic of Korea

^3^Querrey Simpson Institute for Bioelectronics, Northwestern University, Evanston, IL 60208, USA

^4^Department of Materials Science and Engineering, Korea Advanced Institute of Science and Technology, Daejeon 34141, Republic of Korea

^5^Graduate School of Semiconductor Materials and Devices Engineering, Ulsan National Institute of Science and Technology, Ulsan 44919, Republic of Korea

^6^Department of Semiconductor Engineering, Pukyong National University, Busan 48513, Republic of Korea

^7^Department of Materials Science and Engineering, University of California, Davis, California 95616, USA

^8^RanoM Inc., Research and Development Division, Busan 48513, Republic of Korea

^9^These authors contributed equally: Tae Wan Park, Seunghee H. Cho.

^10^Deceased

*Corresponding author

Yeon Sik Jung Email: [ysjung@kaist.ac.kr](mailto:ysjung@kaist.ac.kr)

Woon Ik Park Email: [thane0428@pknu.ac.kr](mailto:thane0428@pknu.ac.kr)

Keywords: Multi-material, nanotransfer printing, nanostructures, crossbar, SERS

**Note S1. Solvent-thermal nanotransfer printing (ST-nTP): An integration of solvent- and thermal-assisted nanotransfer printing methods.**

ST-nTP is a novel patterning process that integrates the advantages of both solvent-assisted nanotransfer printing (S-nTP) and thermal assisted nanotransfer printing (T-nTP). This approach enables high-resolution, large-area patterning under low-temperature conditions, effectively mitigating the inherent limitations of each individual method.

A solvent-assisted nanotransfer printing (S-nTP) process is established through solvent-assisted control of adhesion strength between sticky layer of adhesive tape and polymer replica film layer. This technique enables high-resolution pattern formation with a wide range of pattern widths from the micrometer scale to sub-10 nm. As a polymer-softening-based transfer printing strategy, it allows stable patterning with minimal physical load on the materials to be printed. However, S-nTP struggles to obtain systematically printed nanoscale pattern structures over the chip scale. The necessity of maintaining uniformity across a large area during the patterning process is constrained by the volatile behavior of solvent vapor, such as rapid temporal changes and high sensitivity to ambient conditions. In addition, quantitative control of the induced solvent vapor amount remains technically challenging, frequently leading to excessive swelling of the polymer film and non-uniform pattern formation.

In contrast, T-nTP is an advanced and scalable process capable of fabricating nanoscale pattern regime over a large-area compared to S-nTP. For successful pattern transfer, this method utilizes a heat-rolling press system that can provide both uniform heat and pressure to the patterning surface. It has attracted considerable attention due to its patternability to achieve wafer-scale patterning, which remains one of the most critical challenges in nanopatterning. Nevertheless, the high-temperature required for the pattern transfer pose limitations to the scalability of the replica material, particularly in terms of layer thickness and intrinsic performance of the material.

By combining the solvent-softening advantage of S-nTP and the mechanical uniformity of T-nTP, the ST-nTP process can systematically generate highly uniform and reproducible ultrafine pattern structures. This synergistic effect of simultaneous solvent and thermal interaction enables reducing process temperature and improving material compatibility, particularly for soft polymers or functional layers that are sensitive to thermal stress. This integrated approach not only improves fidelity of printing patterns but enhances scalability and reliability, thereby offering a promising strategy for next-generation nanofabrication.

**Figure S1. Surface modification of master mold.** A hydroxyl-terminated PDMS brush treatment modifies the surface of the master mold, rendering it hydrophobic with low surface energy.

**Figure S2. Structural integrity of sub-20 nm master mold after multiple uses.** SEM and photograph images of master mold following (a) 50 and (b) 100 usage cycles.

**Figure S3. Adhesion strength of solvent-injected PI tape.** (a) Schematic illustration of the experimental setup for the 90° peel-off test. (b) Comparison of adhesion strengths between pristine and solvent-injected PI tapes. The adhesion strength decreased from 4.38 N to 2.77 N after injection of solvent vapor.

**Figure S4. Transfer-printed sub-20 nm structures via the S-nTP and ST-nTP.** (a) Pattern cracks caused by excessive solvent vapor exposure during the S-nTP process. Due to the inherent limitation in quantitatively controlling the amount of induced solvent vapor, excessive swelling of the polymer film frequently occurs, resulting in non-uniform pattern formation. (b) Well-defined line structures obtained under optimized solvent vapor conditions and uniform thermal injection using the ST-nTP process.

**Figure S5. Comparison of transfer-printed patterns produced by solvent-assisted and solvent-thermal-assisted nTP methods.** (a) Limitation of transfer printing at the chip level when using the solvent injection method. (b) Successful pattern formation over a large-area (4.1 cm × 5.2 cm) achieved *via* the ST-nTP process.

**Figure S6. Temperature dependency of the surfaces of PMMA thin films.** The surfaces of PMMA thin films heated at various temperatures, ranging from 50℃ to 150℃, exhibited microcracks caused by the tension developed within the PMMA layer.

**Figure S7. Temperature-dependency of transfer yields for nanostructures.** (a) Photograph images of transfer-printed nanostructures *via* ST-nTP at various temperatures, ranging from 25℃ to 100℃. (b) Transfer yields obtained through ST-nTP and T-nTP processes. The threshold temperature required for successful transfer printing *via* the ST-nTP process was significantly reduced to half of that required by the T-nTP method. All transfer yields were calculated from each transfer-printed image using Image J software.

**Figure S8. Densified PMMA thin film based on heat injection.** (a) Top-view and (b) cross-sectional SEM images of PMMA thin films before and after thermal annealing at a threshold temperature of 75℃. (c) Thickness variation of both as-cast (25℃) and annealed PMMA thin films at 75℃.

**Figure S9. Key parameters determining the threshold temperature for successful pattern transfer.** (a) Comparison of the effects of various solvents on the threshold temperature. (b) Threshold temperature depending on the polymer film thickness.

**Figure S10. Adhesion strength of heat-injected PI tape.** The adhesion strengths of pristine and heat-injected PI tapes were compared through 90° peeling-off tests. The adhesion strength decreased slightly from 4.37 N for the pristine PI tape to 3.33 N after heat injection at the threshold temperature of 75°C.

**Figure S11. Procedure for the fabrication of sub-20 nm ultrafine master mold.** To fabricate a sub-20 nm master mold for the transfer printing process, a surface-patterned guiding template is required. A positive photoresist is spin-coated onto a Si substrate, followed by exposure to deep ultraviolet light with a wavelength of 248 nm. The developed PR patterns, which remain as the inverse shape of the photomask, are used as an etch mask to engrave pattern structures with CF_4_ plasma etching. After the remaining PR layer is removed, the surface-engraved guiding template is formed. Then, the cylinder-forming PS-*b*-PDMS BCP is coated and undergoes solvothermal annealing on the guiding template. The annealed BCP thin film is etched using CF_4_ and O_2_ plasma treatments to remove the top-PDMS and PS matrix, respectively, resulting in sub-20 nm SiO*_x_* line structures.

**Figure S12. Self-assembled SD45 BCP within the guiding template.** (a) Schematic illustration of the cylinder-forming SD45 BCP. (b) Well-defined sub-20 nm SiO*_x_* lines within a guiding template with a width/space of 1 μm/250 nm. (c) GISAXS pattern and (d) 1D GISAXS profile collected from the master mold.

**Figure S13. Reliability studies of transfer-printed sub-20 nm multi-material line structures *via* the ST-nTP.** (a) SEM images and quantitative pattern roughness analysis results of the transfer-printed multi-material pattern structures (Pt, GST, SiO*_x_*, and AlO*_x_*). The mold was analyzed using the SEM image shown in Figure. S8b. (b) Line width deviation between mold and transfer-printed nanostructures. Power spectral density (PSD) analysis for (c) line width roughness and (d) line edge roughness of sub-20 nm mold, Pt, GST, AlO*_x_*, and SiO*_x_* lines. All analytical procedures were conducted using a commercial lithography pattern analysis software (SuMMIT, V13.0.0).

**Figure S14. Deposition strategy for fabricating high-resolution crossbar architectures.** Functional materials must be deposited onto both (a) horizontally and (b) vertically arranged replica patterns to achieve a 90° angle transfer relative to each other.

**Figure S15. EDS elemental mapping images of TiO*_x_*/Pt crossbar arrays.** The EDS mapping results clearly show the high-density TiO*_x_*/Pt crossbar nanoarchitectures with sub-20 nm width.

**Figure S16. Durability of transferred multilayer pattern structures.** (a) Schematic illustration of the experimental setup for the ultrasonic delamination study. (b) SEM images of 3D hierarchically stacked Pt lines with sub-20 nm line width, captured before and after the high-power sonication.

**Figure S17. Multi-material formation on the replica pattern.** (a) Procedure for forming various materials on the replica pattern using partially opened shadow masks. (b) Photographic image of selectively deposited multi-material on the replica pattern.

**Figure S18. (Left) Cross-sectional TEM image of metallic crossbar structure formed on a metal thin film for morphology comparison. (Right) Raman spectra of 10-4 M R6G from 3D Ag crossbar array, Ag nanowire array, and Ag thin film.**

**Figure S19. High-resolution printability of metal nanowires fabricated by ST-nTP.** The distribution of center-to-center spacing of metal nanowires.

**Figure S20. Top-view SEM images of Au 3D metal crossbar arrays (a) as-printed, (b) after drop-casting and drying aqueous solutions, and (c) after Raman analysis.**

**Figure S21. Raman spectra of R6G on a SiO_2_ wafer and cross-view SEM image showing the thickness of the R6G solid film.**

**Note S2. Calculation of enhancement factor (EF).**

Determination of the SERS enhancement factors for each material is achieved by comparing the Raman intensity from a single molecule adsorbed on the SERS substrate to that placed on a reference surface^1,2^. The calculation follows **Equation S1**, where $I_{SERS}$ and $I_{Normal}$ are the Raman intensities from the SERS substrate and SiO_2_ wafer, respectively. Rhodamine 6G, which is known for its high Raman cross-section, is selected as the analyte molecule for this purpose and the Raman intensity is compared at the characteristic Raman band of 1361 cm^-1^.

$EF=\frac{I_{SERS}\times N_{Film}}{I_{Normal}\times N_{SERS}}$	(Equation S1)

$N_{Film}=\frac{hgN_{A}}{M}$	(Equation S2)

$N_{SERS}=\frac{cN_{A}V}{A}$	(Equation S3)

In order to obtain the Raman intensity from a single molecule, $I_{SERS}$ and $I_{Normal}$ are divided by $N_{SERS}$ and $N_{Film}$, respectively. $N_{Film}$ is calculated based on **Equation S2**, where $h$ is the measured thickness of the R6G film formed on the SiO_2_ wafer (822.37 nm) (**Figure S21**), $g$ is the density of solid R6G (1.26 g/cm^3^), $N_{A}$ is the Avogardo’s number, and $M$ is the molecular weight of R6G (479.02 g/mol). $N_{SERS}$ is derived from **Equation S3**, where $c$ and $V$ are the concentration (10^-6^ M) and volume (10 μl), respectively of the R6G solution prepared in ethanol that is drop-cast on to a SERS substrate with an area of $A$ (100 mm^2^). The calculated EF values are shown in **Table S3**.

**Figure S22. Multiplex Raman spectra of mixed sample of 10-4 M R6G, 10^-5^ M TAMRA, and 10^-5^ M cortisol from each material region of Ag, Au, Pt, and Cu in the 3D multi-metal crossbar array.**

**Figure S23. Array of spectra showing SERS spectra from R6G, obtained from 20 points for each material.** (a) Cu, (b) Pt, (c) Au, and (d) Ag.

**Figure S24. Relative standard deviation (RSD) calculated from SERS spectra of R6G using intensities at the characteristic Raman band 1361 cm^-1^ for (a) Cu, (b) Pt, (c) Au, and (d) Ag.**

**Figure S25. SERS spectra of *E. coli* obtained from each material region of the 3D multi-metal array labeled with characteristic Raman bands.** (a) Cu, (b) Pt, (c) Au, and (d) Ag.

**Figure S26. Array of spectra showing SERS spectra from *E. coli*, obtained from 20 points for each material.** (a) Cu, (b) Pt, (c) Au, and (d) Ag.

[ ]: References in Supplementary Information

**Table S1. Comparison of the advantages and limitations between previously reported nTP methods and the ST-nTP process.**

| Material | Wavenumber (cm^-1^) |
| --- | --- |
| Ag | 791, 989, 1127, 1165, 1209, 1236, 1290, 1322, 1356, 1408, 1489, 1558, 1586, 1611 |
| Au | 989, 1165, 1236, 1322, 1356, 1408, 1489, 1558, 1586, 1611 |
| Pt | 1133, 1195, 1228, 1336, 1418, 1492, 1535 |
| Cu | 782, 827, 884, 1021, 1073, 1342, 1441, 1504, 1590, 1615 |

**Table S2. Characteristic peak positions (cm^-1^) of E. coli for different plasmonic materials as SERS substrates.**

| Material | Cu | Pt | Au | Ag |
| --- | --- | --- | --- | --- |
| Enhancement factor  of 10^-9^ M R6G | 6.23×10^5^ | 7.59×10^5^ | 4.71×10^6^ | 1.24×10^7^ |
| Enhancement factor  of 10^-6^ M R6G | 1.60×10^3^ | 5.37×10^3^ | 3.58×10^4^ | 9.57×10^4^ |

**Table S3. Enhancement factor values for Cu, Pt, Au, and Ag of the 3D multi-metal crossbar array.**

**Supplementary References**

1	Le Ru, E. C., Blackie, E., Meyer, M., & Etchegoin, P. G. Surface enhanced Raman scattering enhancement factors: a comprehensive study. *J. Phys. Chem. C.* **111**, 13794-13803 (2007).

2	Wang, S. Y. *et al*. Reactive ion etching-assisted surface-enhanced Raman scattering measurements on the single nanoparticle level. *Appl. Phys. Lett.* **104**, (2014).

3	Sim, K. *et al.* High fidelity tape transfer printing based on chemically induced adhesive strength modulation. *Sci. Rep.* **5**, 16133 (2015).

4	Jeong, J. W. *et al.* High-resolution nanotransfer printing applicable to diverse surfaces via interface-targeted adhesion switching. *Nat. Commun.* **5**, 1-12 (2014).

5	Gan, Z. *et al.* High-fidelity and clean nanotransfer lithography using structure-embedded and electrostatic-adhesive carriers. *Microsyst. Nanoeng.* **9**, 8 (2023).

6	Tiefenauer, R. F., Tybrandt, K., Aramesh, M. & Voros, J. Fast and versatile multiscale patterning by combining template-stripping with nanotransfer printing. *ACS Nano* **12**, 2514-2520 (2018).

7	Sadri, B. *et al.* Wearable and implantable epidermal paper-based electronics. *ACS Appl. Mater. Interfaces* **10**, 31061-31068 (2018).

8	Park, T. W. *et al.* Thermally assisted nanotransfer printing with sub–20-nm resolution and 8-inch wafer scalability. *Sci. Adv.* **6**, eabb6462 (2020).
